# Altered temporal organization of neural response dynamics during attention processing differentiates ADHD subtypes in children

**DOI:** 10.1016/j.nicl.2026.104011

**Published:** 2026-05-23

**Authors:** Yanan Zhang, Tongxia Li, Jun Jiang, Hao Li, Ruidi Sun, Yunjie Li, Wenqi Chen

**Affiliations:** a School of Medicine, Jianghan University, Wuhan, China; b Division of Child Healthcare, Department of Pediatrics, Tongji Hospital, Tongji Medical College, Huazhong University of Science and Technology, Wuhan, China; c Department of Neurology, Tongji Hospital, Tongji Medical College, Huazhong University of Science and Technology, Wuhan, China; d Brain-Computer Interface Research Institute, Tongji Hospital, Tongji Medical College of Huazhong University of Science and Technology, Wuhan, China; e Department of Electrophysiology, Wuhan Children’s Hospital, Tongji Medical College, Huazhong University of Science and Technology, Wuhan, China; f Innovation Center for Brain Medical Sciences, The Ministry of Education of the People's Republic of China, Huazhong University of Science and Technology, Wuhan, China

**Keywords:** Attention-deficit/hyperactivity disorder, EEG neural dynamics, Temporal organization, Neural trajectory analysis, Data-driven neural states, ADHD subtypes

## Abstract

**Background:**

Attention-deficit/hyperactivity disorder (ADHD) shows marked heterogeneity, and conventional event-related potential (ERP) measures have limited sensitivity to subtype differences. This study examined whether alterations in the temporal organization of neural responses during attentional processing differentiate ADHD subtypes.

**Methods:**

Children with predominantly inattentive ADHD (ADHD-I), combined-type ADHD (ADHD-C), and typically developing (TD) controls completed an auditory oddball task during electroencephalography. Neural responses were analyzed using time-resolved scalp topographies, low-dimensional neural trajectory analysis, and data-driven neural state modeling. Associations with clinical symptoms were examined.

**Results:**

Both ADHD subtypes showed altered temporal alignment of neural responses relative to TD children, particularly during target processing. Neural trajectories exhibited reduced differentiation between standard and target stimuli, with ADHD-I showing reduced trajectory separation and ADHD-C showing exaggerated but inefficient state excursions. Data-driven analyses further revealed subtype-specific alterations in neural state stability and transitions, which showed exploratory associations with attentional and behavioral impairment.

**Conclusions:**

ADHD is characterized by disrupted temporal organization of neural responses that is not captured by conventional ERP measures. Subtype-specific neural dynamics provide a mechanistic account of ADHD heterogeneity.

## Highlights

• Time-resolved neural dynamics reveal disrupted temporal organization of attentional processing in ADHD.
• Low-dimensional neural trajectory analysis dissociates inattentive and combined ADHD subtypes.
• ADHD-I shows reduced state differentiation, whereas ADHD-C exhibits exaggerated yet inefficient excursions.
• Neural state dynamics show exploratory links to attentional symptoms and functional impairment..

## 1 Introduction

Attention-deficit/hyperactivity disorder (ADHD) is a prevalent neurodevelopmental condition characterized by persistent difficulties in attentional regulation, impulse control, and goal-directed behavior (Hinshaw, 2018). Beyond its core symptoms, ADHD exhibits pronounced heterogeneity across clinical presentation, cognitive profiles, and functional outcomes (Tripp and Wickens, 2009). This heterogeneity is formally reflected in diagnostic subtypes, most prominently the predominantly inattentive type (ADHD-I) and the combined type (ADHD-C) Association, 2013. However, whether these clinically defined subtypes correspond to dissociable neurophysiological mechanisms remains an open and unresolved question.

Neuroimaging studies have consistently implicated large-scale brain networks in ADHD, including frontoparietal control systems, attentional networks, and the default mode network (DMN), as well as their competitive and cooperative interactions Koirala et al., 2024, Duffy et al., 2017. Aberrant engagement and coordination among these systems have been proposed as central mechanisms underlying attentional lapses, impulsivity, and executive dysfunction in ADHD. Yet, most existing evidence is derived from static or time-averaged measures of brain activity, providing limited insight into how subtype-specific abnormalities unfold over time during stimulus processing. As a result, the temporal dimension of neural processing—arguably critical for attentional control—has remained underexplored in relation to ADHD heterogeneity.

Electroencephalography (EEG) and event-related potentials (ERPs) offer millisecond-level temporal resolution and have long been used to probe attentional and inhibitory processes in ADHD (Xu et al., 2025). In particular, the N200 and P300 components elicited in oddball paradigms are widely interpreted as markers of response inhibition, conflict monitoring, and attentional resource allocation Kaiser et al., 2020, Downes et al., 2017. Although numerous studies have reported altered amplitudes, delayed latencies, or increased trial-to-trial variability of these components in ADHD, findings have been inconsistent across studies and subtypes. Importantly, conventional ERP analyses rely on predefined time windows and component-centric interpretations, implicitly assuming that neural responses follow a relatively fixed temporal structure across individuals and conditions.

Growing evidence from systems and network neuroscience challenges this assumption. Cognitive functions such as attention, decision-making, and inhibitory control are increasingly understood as emerging from the dynamic coordination of distributed neural networks, whose configurations evolve over tens to hundreds of milliseconds (Angelaki et al., 2025). From this perspective, neurodevelopmental disorders may not be adequately characterized by uniform increases or decreases in response magnitude, but rather by disruptions in the temporal organization, sequencing, and stability of neural states (Bochet et al., 2021). In attentional tasks such as the oddball paradigm, the brain must continuously reconfigure between processing frequent, task-irrelevant stimuli and detecting and responding to infrequent targets—a process that places strong demands on the timing and coordination of large-scale neural dynamics.

Accordingly, ADHD-related abnormalities may be more accurately captured by examining how neural response configurations evolve over time, rather than by focusing exclusively on peak amplitudes at isolated latencies. Alterations in the trajectory, separation, or transition structure of neural states could provide a mechanistic account of why individuals with ADHD often achieve comparable behavioral performance while relying on less efficient or more variable neural processes. Moreover, such a dynamic framework offers a principled way to interrogate subtype-specific differences, which may reflect distinct modes of dysregulation in neural state coordination rather than uniform deficits within a single network.

In the present study, we investigated whether alterations in the temporal organization of neural response dynamics during attention processing can differentiate ADHD subtypes. Children with ADHD-I, ADHD-C, and typically developing (TD) controls performed an auditory oddball task while EEG was recorded. Beyond conventional ERP analyses, we applied time-resolved, data-driven approaches to characterize whole-brain neural dynamics, including low-dimensional neural trajectory analysis and state-based modeling of scalp topographies. We hypothesized that ADHD subtypes would exhibit distinct abnormalities in the geometry and separation of neural response trajectories for standard versus target stimuli, and these differences would be reflected in altered stability and transition patterns among dominant neural states. By adopting a dynamic systems perspective, this work aims to provide a mechanistic account of ADHD heterogeneity grounded in the temporal coordination of large-scale brain activity.

## 2 Methods and materials

### 2.1 Participants

A total of 175 children aged 6–14 years were recruited for this study, including TD controls and children diagnosed with ADHD. All ADHD diagnoses were established by experienced clinicians according to DSM-5 criteria. ADHD subtypes were defined using standardized symptom thresholds: children were classified as ADHD-I if inattentive symptom scores were ≥ 6 and hyperactive/impulsive scores were < 6, and as ADHD-C if both inattentive and hyperactive/impulsive symptom scores were ≥ 6. Symptom severity and functional impairment were further assessed using the Chinese versions of the SNAP-IV (Swanson et al., 2001), Conners’ Parent Symptom Questionnaire (PSQ) (Conners et al., 1998), and the Weiss Functional Impairment Rating Scale (WFIRS) (Weiss et al., 2018). An a priori statistical power analysis was conducted using G*Power 3.1 to determine the minimum sample size required for detecting group differences. For a one-way ANOVA with three groups, assuming a medium effect size (Cohen’s f = 0.25), a significance level of α = 0.05, and a statistical power of 0.80, the required total sample size was estimated to be 159 participants. The final dataset included 175 participants, exceeding this requirement and therefore providing adequate statistical power for the main analyses.

All participants were right-handed, medication-naïve with respect to ADHD-related treatments, and had a full-scale IQ > 60 as assessed by the Wechsler Intelligence Scale-IV(WISC-IV) for Children. Children with comorbid epilepsy, a history of traumatic brain injury, severe psychiatric conditions (e.g., major depression), or current use of ADHD medication were excluded to minimize potential confounding neurodevelopmental factors. Written informed consent was obtained from parents or legal guardians, and verbal assent was obtained from the children. The study protocol was approved by the Clinical Research Ethics Committee of Wuhan Children’s Hospital (2024R009-E03) and conducted in accordance with the Declaration of Helsinki.

Demographic, behavioral, and clinical characteristics of the three groups are summarized in Table 1. Due to technical issues during data acquisition, behavioral accuracy (ACC) and reaction time (RT) data were unavailable for a small number (6 TD children, 1 ADHD-I child, and 4 ADHD-C children) of participant. Despite the missing behavioral logs, the EEG data for these 11 participants were retained in the final analyses. Active task engagement for these children was confirmed by their successful completion of the pre-recording practice session and the presence of distinct target-related ERP components (e.g., P300) at the single-subject level.

**Table1:** Characteristics of study participants.

Characteristic		TD	ADHD-I	ADHD-C	Statistic		
Subject		n = 71	n = 62	n = 42			
Gender (M/F)		52/19	44/18	32/10	*P* = 0.8402 (Chi-square test)		

		**Mean ± SD**			**P-value (Kruskal-Wallis test with Dunn- Šidák comparisons test)**		
					ADHD-I & TD	ADHD-C & TD	ADHD-I & ADHD-C
Age(years)		9.01 ± 1.86	9.59 ± 2.09	8.74 ± 1.88	0.5319	0.3303	0.9629
IQ		88.20 ± 14.55	91.37 ± 11.67	92.85 ± 12.69	0.2998	0.8511	0.1069
RT (ms)		579.09 ± 128.55	539.4 ± 137.61	584.23 ± 139.64	0.1813	0.9973	0.2130
ACC (%)		98.08 ± 3.71	98.73 ± 1.68	97.72 ± 3.78	1.0000	0.8170	0.8429
Standard trial number		278.87 ± 3.71	273.21 ± 1.68	277.29 ± 3.78	0.5068	0.9993	0.5428
Target trial number		81.07 ± 128.55	77.85 ± 137.61	78.74 ± 139.64	0.0022	0.0743	0.8343
SNAP-IV	Attention deficit	1.12 ± 0.46	1.74 ± 0.6	2.03 ± 0.62	1.95E-08	1.43E-11	0.2512
	Hyperactive	0.68 ± 0.5	0.87 ± 0.55	1.7 ± 0.53	0.1695	2.43E-14	9.18E-09
Conners	Conduct problem	0.62 ± 1.06	0.76 ± 0.4	1.05 ± 0.52	0.0008	1.57E-08	0.0364
	Learning problem	1.15 ± 0.63	1.77 ± 0.64	1.84 ± 0.6	2.49E-07	1.05E-07	0.8582
	Psychosomatic	0.27 ± 0.32	0.43 ± 0.43	0.48 ± 0.56	0.1104	0.2878	0.9938
	Impulsive-Hyperactive	0.73 ± 0.64	0.9 ± 0.41	1.37 ± 0.63	5.29E-02	1.77E-08	0.0009
	Anxiety	0.35 ± 0.38	0.57 ± 0.58	0.56 ± 0.52	9.46E-02	0.1345	0.9998
	Hyperactivity index	0.73 ± 0.31	1.14 ± 0.38	1.42 ± 0.49	3.18E-08	1.69E-13	0.0559
Weiss	Family	0.32 ± 0.26	0.6 ± 0.34	0.82 ± 0.48	1.71E-05	6.37E-09	0.1674
	School and learning	0.59 ± 0.34	0.9 ± 0.42	0.85 ± 0.51	0.0002	0.0167	0.8158
	Life skills	0.51 ± 0.33	0.9 ± 0.39	1 ± 0.52	1.13E-07	5.62E-08	0.8660
	Child's self-concept	0.53 ± 0.5	0.77 ± 0.56	0.85 ± 0.66	0.0271	0.0135	0.9446
	Social activities	0.37 ± 0.36	0.62 ± 0.35	0.7 ± 0.46	7.88E-05	5.06E-05	0.9324
	Risky activities	0.18 ± 0.17	0.25 ± 0.19	0.37 ± 0.26	0.1476	3.43E-05	0.0291

### 2.2 Experimental paradigm and EEG recording

Participants completed an auditory oddball task while EEG was recorded. Two types of auditory stimuli were presented binaurally via headphones at approximately 65 dB: a standard tone (80% probability) and a target tone (20% probability). Participants were instructed to respond to target stimuli with a button press and to withhold responses to standard stimuli. Prior to the formal recording, participants completed a brief practice session to ensure task comprehension.

EEG data were acquired using a 21-channel system (NSW2, Neuracle, Changzhou, China) with Ag/AgCl electrodes placed according to the international 10–20 system. All electrode impedances were maintained below 10 kΩ. Signals were sampled at 1000 Hz and referenced online to the recording system default.

### 2.3 EEG preprocessing

EEG preprocessing was performed in MATLAB R2024b using EEGLAB (Delorme and Makeig, 2004). Data from mastoid electrodes (A1 and A2) were removed, and signals were downsampled to 500 Hz and re-referenced to the common average. Continuous data were band-pass filtered between 0.1 and 100 Hz. Artifacts related to eye movements, muscle activity, and cardiac signals were identified and removed using independent component analysis.

The cleaned EEG data were segmented into epochs spanning from −200 ms to 800 ms relative to stimulus onset. Baseline correction was applied using the pre-stimulus interval. Epochs with amplitudes exceeding ± 500 μV were rejected to remove rare extreme artifacts (e.g., electrode detachment or cable movement). For each participant, artifact-free epochs were averaged separately for standard and target stimuli to obtain condition-specific ERP waveforms.

### 2.4 Conventional ERP analysis

To assess whether classical ERP components captured subtype-specific differences, we quantified the N200 component elicited by standard stimuli (150–250 ms) and the P300 component elicited by target stimuli (250–450 ms). Mean amplitudes and peak latencies were computed across all electrodes. In addition, trial-to-trial stability within these time windows was estimated by calculating inter-trial topographic similarity, providing an index of response consistency.

### 2.5 Time-resolved topographic analysis

To examine the temporal evolution of scalp voltage patterns beyond predefined ERP components, stimulus-locked responses from 0 to 600 ms were divided into six consecutive 100-ms time windows (T1–T6). Within each window, scalp topographies were averaged for each participant and condition. Group-level differences in amplitude and topographic similarity were assessed across time windows to characterize alterations in the temporal organization of neural responses.

### 2.6 Neural trajectory analysis in low-dimensional state space

To characterize large-scale neural dynamics, we applied principal component analysis (PCA) to time-resolved scalp topographies. For each participant, trial-averaged ERP signals for standard and target stimuli within 0–600 ms were downsampled into 100-ms windows and mean-centered across channels. Group-level averages were then subjected to PCA, and neural response trajectories were visualized in the space defined by the first two principal components.

To enable statistical inference, a bootstrap resampling procedure was employed. In each iteration, 15 participants were randomly sampled from each group to construct pseudo-populations, and PCA was repeated 1000 times. Within the resulting state space, we quantified (i) the Euclidean distance between standard and target stimulus trajectories, (ii) the angular divergence between trajectories relative to the initial state (T1) as an index of neural state separation, and (iii) the total trajectory length and inter-timepoint distances as measures of state transition complexity and efficiency.

### 2.7 Identification of dominant neural states and temporal projections

To obtain a unified, data-driven representation of dominant neural response patterns, mean-centered scalp topographies from all groups and both stimulus conditions were concatenated along the temporal dimension and subjected to PCA. The first four principal components (PC1–PC4), together explaining over 95% of the total variance, were retained as group-level dominant neural states.

For each participant, instantaneous scalp topographies at each time point were projected onto these four components, yielding time-resolved projection coefficients that indexed the strength of neural activation along each state. This procedure allowed the ERP responses to be represented as continuous trajectories within a four-dimensional neural state space. Differences between target and standard stimulus projections were computed to isolate attention-related dynamics.

### 2.8 Classification analysis

To evaluate whether dynamic neural features could distinguish ADHD subtypes, a support vector machine classifier (SVM) Meyers, 2013 was trained using the time-resolved projection differences across PC1–PC4. Classification performance for the three groups was assessed using 10-fold cross-validation, repeated 500 times. Chance-level accuracy was estimated via label shuffling, and classification performance was statistically compared against shuffled baselines.

### 2.9 Neural state discretization and transition analysis

To characterize discrete temporal organization, we adopted a microstate-inspired approach. The four PCA-derived scalp topographies served as spatial templates. At each time point, individual scalp configurations were assigned to the PC state with the highest polarity-invariant Pearson correlation. Time points that did not significantly correlate with any PC template were labeled as an “Other” state.

For each participant and condition, state transition matrices were constructed from the resulting state sequences. State stability was quantified as the proportion of self-transitions for each PC state, and inter-state transition probabilities were computed to assess differences in neural state coordination across groups.

### 2.10 Source localization

To provide a coarse anatomical interpretation of the PCA-derived neural states, standardized low-resolution brain electromagnetic tomography (sLORETA) (Pascual-Marqui, 2002) was applied. Source estimates were computed using a minimum-norm inverse solution, yielding standardized current density distributions for each principal component. These results were used solely for qualitative anatomical reference rather than precise localization.

### 2.11 Statistical analysis

Group differences in demographic variables, behavioral measures, ERP metrics, neural trajectory features, and state parameters were assessed using nonparametric Kruskal–Wallis tests followed by Dunn–Šidák post hoc comparisons. Associations between neural dynamic measures and clinical symptom scores were examined using Pearson correlation analysis. Statistical significance was set at *P* < 0.05 for all analyses.

## 3 Results

### 3.1 Limited sensitivity of conventional ERP components to ADHD subtype differences

**Fig. 1:**
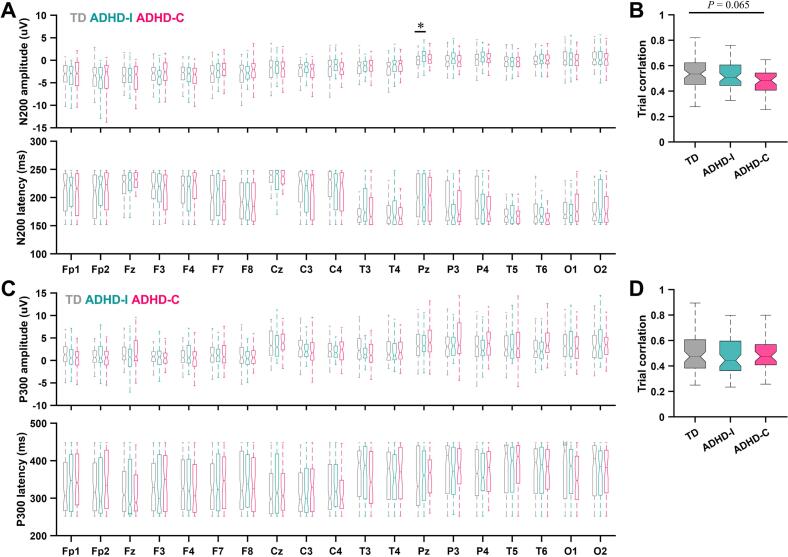
ERP components in the oddball paradigm. (A) N200 amplitude (top) and latency (bottom) across all electrodes during standard-stimulus processing in TD children and ADHD subgroups. The analysis window was 150–250 ms post-stimulus. (B) Trial-wise scalp topography correlations within the N200 time window for TD children and ADHD subgroups. (C–D) Same as (A–B), but for the P300 component elicited by target stimuli, analyzed within 250–450 ms post-stimulus. Boxplots indicate the median (center line), interquartile range (box), and 1.5 × interquartile range (whiskers); outliers are not shown. Group differences were assessed using Kruskal–Wallis tests followed by Dunn–Šidák post hoc multiple comparisons. *P < 0.05.

We first examined whether classical ERP components captured subtype-specific differences during attentional processing. The N200 component elicited by standard stimuli (150–250 ms) and the P300 component elicited by target stimuli (250–450 ms) were quantified across all scalp electrodes (Fig. 1**A and C;** Supplementary Fig. 1). Overall, amplitudes and latencies of both components showed substantial overlap across TD children and the two ADHD subtypes. Apart from a localized increase in N200 amplitude at parietal sites in the ADHD-I group compared with TD children, no robust group differences were observed in either N200 or P300 amplitude or latency distributions.

We further assessed the temporal stability of these components by quantifying trial-to-trial topographic consistency within the corresponding time windows (Arnett et al., 2023). While TD children showed relatively stable N200 and P300 responses, the ADHD-C group exhibited a trend toward reduced N200 stability (Fig. 1**B and D**), consistent with increased variability during early inhibitory processing (Saville et al., 2015). Nevertheless, taken together, conventional ERP measures demonstrated limited sensitivity in differentiating ADHD subtypes, motivating subsequent analyses focused on the temporal organization of neural responses beyond predefined component windows.

### 3.2 Altered time-resolved topographic dynamics during stimulus processing in ADHD

**Fig. 2:**
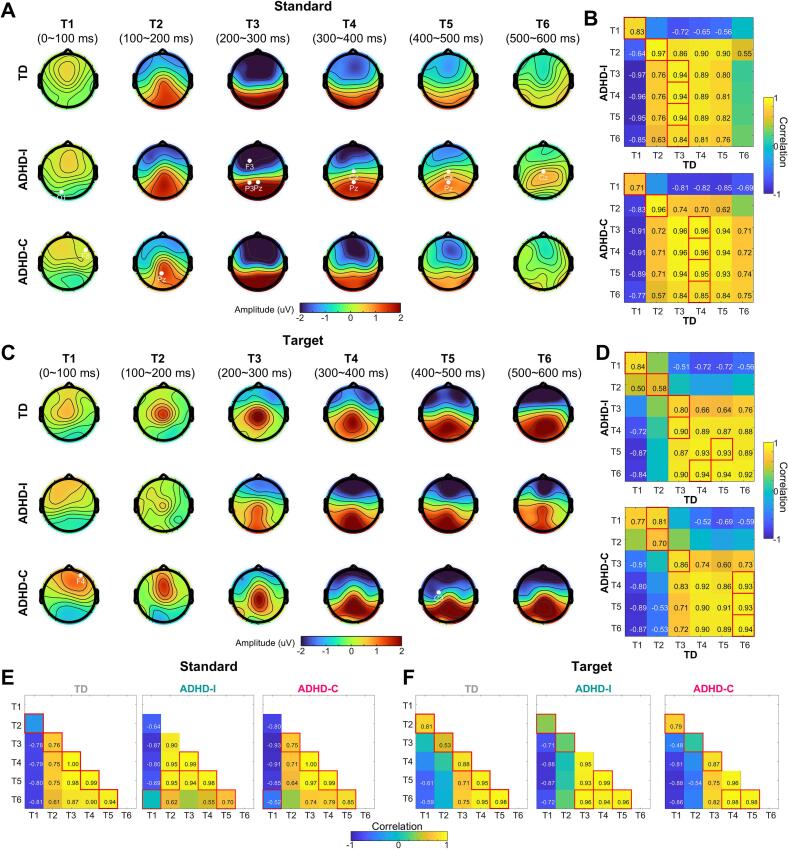
Altered time-resolved topographic dynamics during task performance in ADHD. (A) Whole-brain scalp topographic dynamics during standard-stimulus processing in TD children and ADHD subgroups, averaged within consecutive 100-ms time windows (T1–T6). White markers indicate electrodes showing significant differences relative to the TD group (Kruskal–Wallis test with Dunn–Šidák post hoc correction). (B) Pearson spatial correlations between each ADHD subgroup and the TD group during standard-stimulus processing. Red boxes mark time windows with maximal correlation. Cells without numerical values indicate non-significant correlations (FDR correction for multiple testing). (C–D) Same as (A–B), but for target-stimulus processing: whole-brain topographic dynamics (C) and cross-group spatial correlations (D). (E) Within-group self-correlation matrices during standard-stimulus processing for TD children and ADHD subgroups. Red boxes indicate maximal correlations; non-significant cells are not displayed. (F) Same as (E), but for target-stimulus processing. (For interpretation of the references to colour in this figure legend, the reader is referred to the web version of this article.)

To characterize the temporal evolution of neural responses, scalp topographies from 0 to 600 ms post-stimulus were divided into six consecutive 100-ms windows (T1–T6; Fig. 2**A and C**). Across these windows, both ADHD subtypes exhibited time- and region-specific deviations from TD children. In particular, the ADHD-I group showed enhanced frontoparietal amplitudes during standard-stimulus processing from approximately 200 ms onward (T3–T6), consistent with increased neural engagement during response inhibition.

To assess temporal alignment of response configurations, we first quantified the spatial correlation between each ADHD subgroup and the TD group across time windows. For both standard and target stimuli, the maximal cross-group correlations for ADHD-I and ADHD-C deviated from the diagonal of the correlation matrix (Fig. 2**B and D**), indicating temporal displacement of peak neural similarity relative to TD children. This displacement was more pronounced during target-stimulus processing, where correlations were more diffusely distributed across non-adjacent time windows, suggesting greater temporal dispersion of attentional responses.

We further examined within-group temporal organization by computing self-correlation matrices separately for each group under standard and target conditions. In TD children, maximal correlations consistently aligned along the diagonal for both stimulus types (Fig. 2**E and F**), reflecting a stable and orderly temporal progression of scalp topographies. In contrast, both ADHD subtypes showed systematic shifts of maximal self-correlations away from the diagonal during standard and target processing, indicating disrupted temporal consistency in the evolution of neural response patterns.

Together, these analyses demonstrate that ADHD is associated with altered temporal alignment of large-scale neural responses. Rather than reflecting uniform changes in response magnitude, both ADHD subtypes exhibit mis-timed and more dispersed neural configurations over time.

### 3.3 Divergent neural response trajectories across ADHD subtypes

**Fig. 3:**
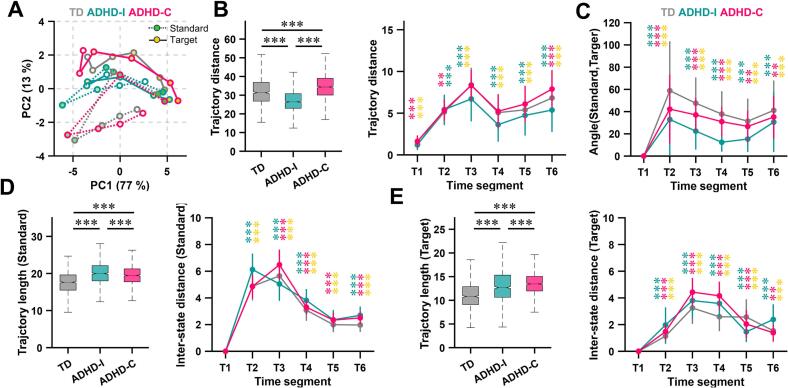
Altered differentiation of neural trajectories in ADHD. (A) Neural response trajectories for standard and target stimuli projected into principal component space for TD children and ADHD subgroups. Responses were averaged within 100-ms time windows (T1–T6). Dashed lines indicate standard-stimulus trajectories, and solid lines indicate target-stimulus trajectories. Color saturation decreases with time progression. (B) Left: total Euclidean distance between group-level trajectories for standard and target stimuli (n = 1000 bootstrap iterations). Right: Euclidean distance between standard- and target-stimulus trajectories at each time window. (C) Angular separation between standard- and target-stimulus trajectories across time, computed as the angle between the trajectory at each time window and the initial time window (T1) in two-dimensional principal component space. (D) Total Euclidean length (left) and inter-window trajectory distance (right) of group-level standard-stimulus trajectories. (E) Same as (D), but for target-stimulus trajectories. Data are presented as boxplots or mean ± standard deviation. Boxplot conventions are as in Fig. 1. Group differences were assessed using Kruskal–Wallis tests with Dunn–Šidák post hoc correction. *P < 0.05, **P < 0.01, ***P < 0.001.

To quantify these dynamic differences, we reconstructed neural response trajectories in a low-dimensional state space defined by principal component analysis (Fig. 3**A,** Supplementary Fig. 2). Standard and target stimuli exhibited distinct geometric structures: standard-stimulus trajectories formed a triangular-like pattern, whereas target-stimulus trajectories approximated a more linear progression, reflecting differences in network engagement between inhibitory and attention-driven processing.

Critically, the separation between standard and target trajectories differed across groups. In the ADHD-I group, trajectories for the two stimulus types overlapped substantially, resulting in significantly reduced inter-trajectory Euclidean distances compared with TD children (Fig. 3**B**). This pattern indicates diminished neural state differentiation between stimulus conditions. In contrast, the ADHD-C group showed greater trajectory distances than TD children, suggesting exaggerated state excursions. However, analysis of trajectory angles revealed that both ADHD subtypes exhibited smaller angular divergence between standard and target trajectories than TD children (Fig. 3**C**), indicating reduced directional separation despite differences in excursion magnitude.

Both ADHD subtypes also displayed significantly longer trajectory lengths during stimulus processing (Fig. 3**D and E**), reflecting greater overall neural state displacement. This effect was condition-dependent: ADHD-I children exhibited longer trajectories during standard stimuli, whereas ADHD-C children showed longer trajectories during target stimuli. These findings suggest subtype-specific inefficiencies in neural state transitions, with increased neural “travel” required to process task demands.

Supplementary analyses were conducted to examine potential age and gender effects on neural trajectory dynamics. No significant linear correlations were observed between age and trajectory length or the state-space distance between Standard and Target trajectories. Age-binned analyses suggested distinct developmental patterns across groups, with TD children showing relatively stable trajectory topology and increasing separation between Standard and Target trajectories, whereas ADHD-I and ADHD-C exhibited divergent trajectory patterns (Supplementary Fig. 3**A**). In addition, gender-subgroup analyses revealed highly similar trajectory topology between male and female participants across all diagnostic groups, indicating that the primary PCA trajectory findings were unlikely to be driven by gender differences (Supplementary Fig. 3**B**).

### 3.4 Data-driven identification of dominant neural states reveals abnormal activation dynamics

**Fig. 4:**
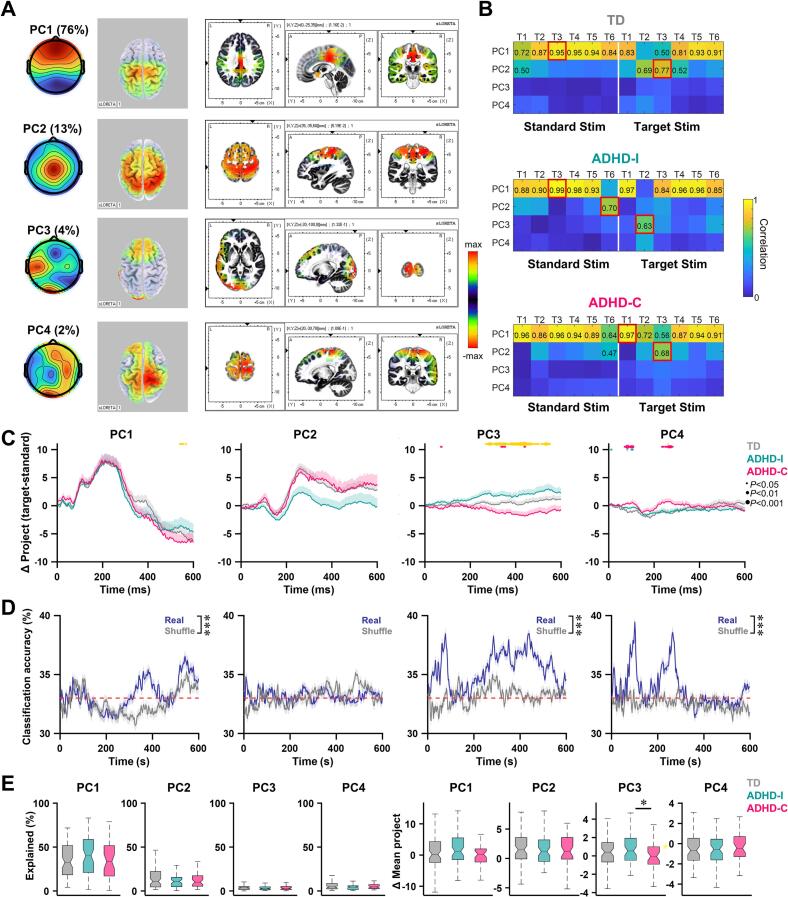
Data-driven extraction of neural states. (A) Four characteristic scalp topographic states and their corresponding sLORETA source localizations, extracted from concatenated group-averaged standard- and target-stimulus responses (0–600 ms) across TD children and ADHD subgroups. Principal component analysis (PCA) was applied to the concatenated responses, and the first four components together explained > 95% of the total variance. (B) Pearson spatial correlations between the four characteristic topographic states and the three groups, with polarity ignored. Red boxes indicate maximal correlations; non-significant cells are not shown (FDR correction for multiple testing). (C) Subject-level differences in projections onto each of the four characteristic topographic states between target and standard stimuli. Colored dots above trajectories indicate significant group differences at the corresponding time windows; dot size reflects significance level (Kruskal–Wallis test with Dunn–Šidák post hoc correction). Green: ADHD-I vs. TD; magenta: ADHD-C vs. TD; yellow: ADHD-I vs. ADHD-C. (D) Support vector machine (SVM) classification based on subject-level projection differences across the four characteristic states. A 10-fold leave-one-out cross-validation was performed and repeated 500 times. Classification accuracy obtained after random label shuffling is shown for comparison. The red dashed line indicates chance-level performance (33%). Differences between real and shuffled accuracies were assessed using a rank-sum test. (E) Explained variance (left) and mean projection differences (right) for each characteristic topographic state. Data in (C–D) are presented as mean ± standard error. Boxplot conventions and statistical tests are as in Fig. 3. *P < 0.05, ***P < 0.001. (For interpretation of the references to colour in this figure legend, the reader is referred to the web version of this article.)

To determine whether these dynamic abnormalities depended on predefined temporal segmentation, we applied PCA to the full 0–600 ms response across all participants and conditions. Four dominant neural states (PC1–PC4) were identified, jointly explaining over 95% of the total variance (Fig. 4**A**). Source reconstruction using sLORETA provided a coarse anatomical context for these states.

PC1 was characterized by conjoint activation of anterior cingulate, parietal, and limbic regions, and showed peak correlation with TD scalp topographies during standard-stimulus processing at T3 (Fig. 4**B**), consistent with cognitive control and conflict monitoring. PC2 reflected widespread frontoparietal activation and peaked during target processing in TD children, aligning with attentional orienting. PC3 and PC4 exhibited left- and right-lateralized patterns, respectively, and showed minimal engagement during canonical TD responses.

Notably, ADHD subtypes displayed distinct alterations in the temporal recruitment of these states. PC3 showed significantly enhanced correlation with scalp topographies during early target processing (T2) in the ADHD-I group, suggesting atypical engagement of this state during attentional selection. Moreover, the maximal spatiotemporal correlations of PC1 and PC2 occurred during standard stimuli in ADHD-I, but during target stimuli in ADHD-C (Fig. 4**B**), indicating subtype-specific shifts in response configuration.

Projection of individual ERP responses into the PC-defined state space revealed marked group differences in target–standard projection contrasts (Fig. 4**C;** Supplementary Fig. 4). A support vector machine classifier trained on these dynamic features achieved above-chance discrimination among the three groups (Fig. 4**D**), despite comparable behavioral performance. Importantly, the variance explained by each PC did not differ across groups (Fig. 4**E**, left), indicating that group differences arose from differential temporal modulation rather than the presence or absence of specific neural states. Quantification of projection differences revealed significant subtype divergence in PC3 activity, with sustained target-related engagement in ADHD-I and reduced engagement in ADHD-C (Fig. 4**E**, right).

## 4 Abnormal neural state stability and transition structure in ADHD

**Fig. 5:**
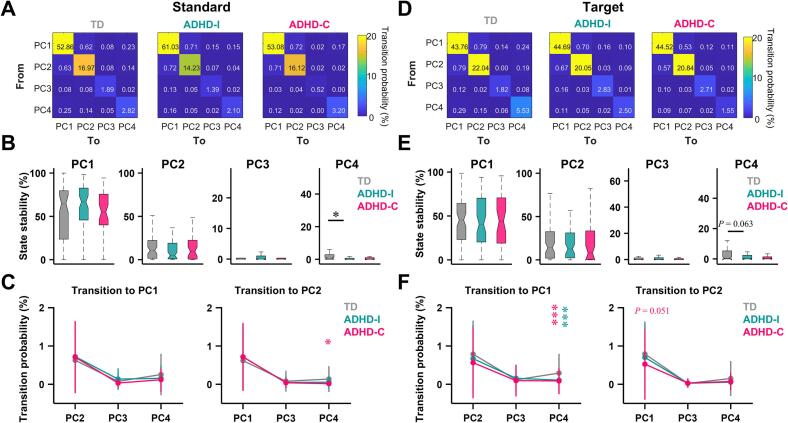
Abnormal neural state transitions in ADHD. (A) Mean state transition matrices during standard-stimulus processing for TD children and ADHD subgroups. (B) State stability (self-transition probability) for each characteristic topographic state during standard-stimulus processing. (C) Transition probabilities from each characteristic state to PC1 (left) or PC2 (right) during standard-stimulus processing. (D–F) Same as (A–C), but for target-stimulus processing. Data are presented as mean ± standard deviation. Boxplot conventions and statistical analyses are as in Fig. 3. *P < 0.05, **P < 0.001.

To further characterize temporal organization at a discrete level, we analyzed neural state sequences using a microstate-inspired approach. State transition matrices revealed pronounced group differences during both standard and target processing (Fig. 5**A and D**). In ADHD-I, the self-transition probability of PC4 was significantly reduced relative to TD children, indicating decreased state stability (Fig. 5**B and E**).

We examined transitions involving PC1 and PC2, given their putative roles in inhibitory control and attentional orienting. During target processing, both ADHD subtypes exhibited significantly reduced transitions from PC4 to PC1 (Fig. 5**F**), suggesting impaired switching from right-biased intermediate state to higher-order control states. Additionally, the ADHD-C group showed reduced transitions from PC4 to PC2 during standard processing, and a trend toward reduced PC1-to-PC2 transitions during target processing, indicating more pronounced disruption of state coordination in this subtype.

We additionally analyzed the temporal proportion of the residual Other state and its transition dynamics. Across all groups, the Other state accounted for approximately 20% of total time points, and no significant differences were observed among the TD, ADHD-I, and ADHD-C groups. This indicates that the group differences identified in the dominant neural states were unlikely to be driven by unequal proportions of unclassified activity. However, within the ADHD-I group, the proportion of the Other state was significantly higher in the Target condition compared with the Standard condition, suggesting increased neural variability under higher task demands in this subtype. In addition, transition analysis revealed that during the Standard condition, ADHD-I exhibited a relatively increased transition probability from PC4 to the Other state compared with the TD group (Supplementary Fig. 5).

### 4.1 Associations between neural dynamics and functional impairment

**Fig. 6:**
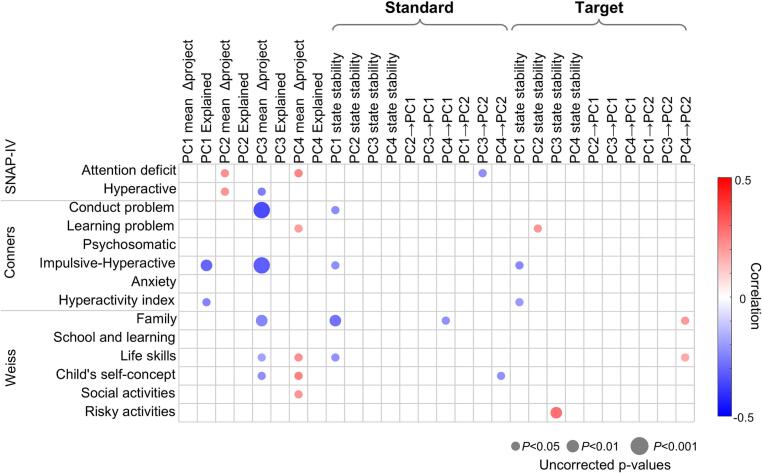
Associations between neural dynamics and clinical measures in ADHD. Correlation matrix illustrating relationships between neural dynamic metrics and clinical measurements within the ADHD group. To control for potential demographic confounds, partial correlation analyses controlling for Age and IQ were additionally performed. Note: The p-values reported are uncorrected, none of the displayed correlations survived FDR correction.

Finally, we explored associations between neural dynamic features and clinical measures within the ADHD groups (Fig. 6). Reduced stability and variance explained by PC1 state were negatively correlated with hyperactive–impulsive symptoms, behavioral problems, and family dysfunction, consistent with a role for impaired control-related dynamics in functional impairment. In contrast, exaggerated target-related projection differences in PC2 and PC4 were positively associated with attentional deficits and broader functional difficulties, including learning problems and reduced life skills.

Interestingly, enhanced PC3 activity during target processing was negatively correlated with hyperactive symptoms, as well as psychosomatic complaints and family difficulties, suggesting a potential compensatory role for this state. In contrast, a higher probability of PC3 self-transitions during target processing showed a positive association with engagement in risky activities, which may indicate that such compensatory dynamics are accompanied by less optimal behavioral tendencies. It should be noted that, the cross-sectional nature of the study precludes causal inference, and neural state dynamics and behavioral traits may simply co-occur. Moreover, none of these clinical correlations survived false discovery rate (FDR) correction for multiple comparisons. Consequently, these findings are considered exploratory and suggest preliminary trends rather than definitive brain-behavior relationships.

## 5 Discussion

In the present study, we investigated whether alterations in the temporal organization of neural responses during attentional processing provide a mechanistic account of neurophysiological heterogeneity in ADHD. By integrating conventional ERP measures with time-resolved topographic analysis and data-driven neural state modeling, we demonstrate that ADHD-related abnormalities are more robustly expressed in the dynamics and coordination of neural states than in the magnitude or latency of canonical ERP components. Importantly, distinct patterns of dynamic reorganization differentiated the inattentive and combined subtypes, despite largely comparable behavioral performance.

### 5.1 ADHD-related abnormalities are expressed in temporal organization rather than ERP magnitude

The N200 and P300 components are widely regarded as canonical ERP markers associated with inhibitory control and attentional processing. However, in the present study, these traditional ERP measures did not consistently reveal robust group differences between ADHD subtypes and typically developing children (Fig. 1). This finding is not unexpected, as previous studies have also reported no significant differences in P300 amplitude or latency in children with ADHD (Xu et al., 2025, Rothenberger et al., 2000). A recent meta-analysis found that although ADHD participants tend to show reduced cue-P300 amplitudes and prolonged Go-P300 latencies, the overall effect sizes were moderate and substantial heterogeneity across studies was observed (Kaiser et al., 2020). One possible explanation is the pronounced heterogeneity within ADHD populations, where variability in symptom profiles, developmental stages, and cognitive strategies may obscure group-level differences when using conventional single-component ERP metrics (Peisch et al., 2021, Liotti et al., 2007, Petersen et al., 2018). Neural processes underlying cognitive control are likely to involve distributed and dynamic population-level activity patterns that may not be fully captured by isolated ERP components. Emerging evidence also suggests that single-trial variability in ERP responses may provide more sensitive indicators of neural dysfunction than traditional averaged ERP measures (Arnett et al., 2023). In this context, the neural trajectory analysis adopted in the present study provides a complementary perspective by characterizing the temporal evolution of neural population dynamics in a low-dimensional state space.

Our results suggest that ADHD-related neural dysfunction may not primarily manifest as deficits in response generation per se, but as alterations in how neural responses are organized and sequenced over time. Children with ADHD were able to recruit sufficient neural resources to perform the task, yet the trajectories and transitions through neural state space were markedly altered (Castellanos and Proal, 2012). This dissociation between behavioral output, ERP amplitude, and underlying neural dynamics underscores the limitations of component-centric analyses for capturing disorder-related heterogeneity.

From a systems-level perspective, attentional processing depends on the coordinated evolution of large-scale networks across time. The oddball paradigm requires continuous switching between suppression of frequent, task-irrelevant stimuli and rapid engagement of attentional and control processes in response to infrequent targets. Our findings indicate that this temporal coordination is disrupted in ADHD, leading to inefficient or poorly differentiated neural response patterns even when overt task performance is preserved.

### 5.2 Altered neural trajectories reveal inefficient and subtype-specific network dynamics

Low-dimensional neural trajectory analysis provided a compact geometric representation of these dynamic abnormalities (Perich et al., 2025). In TD children, standard and target stimuli occupied well-separated trajectories with distinct directions in state space, reflecting efficient differentiation between inhibitory and attentional processing modes (Fig. 2, Fig. 3). In contrast, both ADHD subtypes exhibited reduced directional separation between trajectories, suggesting impaired differentiation of neural states across task demands.

Notably, the two ADHD subtypes diverged in the manner of this impairment. ADHD-I was characterized by reduced spatial separation between standard and target trajectories, indicating a failure to establish distinct neural configurations for different stimulus contexts (Zhang et al., 2025, Sripada et al., 2014). ADHD-C, in contrast, showed exaggerated trajectory excursions but similarly reduced directional separation, implying greater neural effort without commensurate gains in functional differentiation (Fassbender and Schweitzer, 2006). These findings align with models proposing inefficient or compensatory recruitment of control-related networks in ADHD, particularly in the combined subtype (Qian et al., 2019).

The increased trajectory length observed in both subtypes further supports the notion of inefficient neural processing. Longer trajectories imply that neural activity traverses a greater distance in state space to accomplish comparable task demands, potentially reflecting redundant or unstable transitions between network configurations. Such “detours” in neural dynamics may contribute to the well-documented susceptibility of children with ADHD to cognitive fatigue and performance variability during sustained tasks (Mullins et al., 2005). These results suggest that although behavioral accuracy and reaction times were comparable across groups, similar task performance may arise from different neural processing strategies, potentially reflecting compensatory mechanisms whereby children with ADHD achieve comparable outcomes through alternative or less efficient neural dynamics.

TD children showed features consistent with normative network maturation, characterized by relatively stable trajectory topology and a tendency toward increased neural differentiation with age, in line with evidence that large-scale brain networks become progressively more specialized during development. In contrast, the ADHD subgroups appeared to exhibit atypical trajectories, with the ADHD-C group showing pronounced topological instability across age bins, potentially reflecting fluctuating recruitment of task-related neural configurations. Compared with the developmental effects of age, the fundamental spatial organization of these neural dynamics appeared largely robust to gender differences. Although female participants in the ADHD subgroups showed a preliminary tendency toward greater trajectory length and state-space distance—possibly indicating increased compensatory cognitive engagement—this observation should be interpreted cautiously given the limited female sample size (Supplementary Fig. 3**).**

### 5.3 Disrupted neural state stability and transitions underlie attentional dysregulation

Beyond continuous trajectories, discrete state analysis revealed abnormalities in the stability and transition structure of dominant neural states. States associated with cognitive control (Li et al., 2021, Menon and D’Esposito, 2022) (PC1) and attentional orienting (Markett et al., 2022, Griffiths et al., 2021) (PC2) were present in all groups and explained comparable proportions of variance, indicating that ADHD is not characterized by the absence of specific neural states (Fig. 4, Fig. 6). Instead, group differences emerged in how these states were temporally recruited, maintained, and transitioned between.

All ADHD subtypes exhibit impaired transition capacity from the right-dominant frontoparietal network (Silk et al., 2005) (PC4) to higher-order control networks (PC1), suggesting a shared deficit in coordinating bottom-up and top-down processes (Nee, 2021). Subtype-specific patterns further emerged, with ADHD-C exhibiting more pronounced impairments in transitions involving attentional orienting states. These findings provide a dynamic account of why ADHD-C is often associated with heightened impulsivity and attentional instability: neural states may be recruited but fail to transition efficiently into configurations that support sustained, goal-directed behavior.

### 5.4 Potential compensatory dynamics and their behavioral implications

Notably, several neural dynamic metrics showed exploratory associations with behavioral rating scales of attentional and executive function impairments, indicating that these electrophysiological measures may capture clinically meaningful variations in cognitive functioning. An intriguing finding was the differential role of a left-lateralized neural state (PC3) during target processing. Enhanced engagement of this state in ADHD-I was associated with reduced symptom severity and functional impairment, suggesting a potential potential compensatory correlate. This interpretation is consistent with prior evidence linking left fronto-temporal connectivity (Arrington et al., 2019) to improved attentional control following cognitive training (Liu et al., 2024). However, excessive stability of this state was also associated with increased engagement in risky behaviors, indicating that compensatory dynamics may carry trade-offs and are not uniformly beneficial (Fig. 6).

Together, these results highlight that neural dynamics in ADHD cannot be reduced to uniformly deficient or hyperactive processes. Instead, ADHD appears to involve a reorganization of neural state coordination, in which compensatory recruitment may partially offset deficits in one domain while introducing vulnerabilities in others.

### 5.5 Methodological implications and relation to existing models

Methodologically, our findings demonstrate the added value of dynamic, data-driven approaches for studying neurodevelopmental disorders (Michel and Koenig, 2018, Buzzell et al., 2022, Brunton et al., 2016). By moving beyond predefined ERP components and embracing a state-space framework, we were able to capture latent organizational principles of neural activity that are invisible to conventional analyses. This approach complements existing models of ADHD that emphasize default mode interference or frontoparietal dysfunction, by explicitly incorporating the temporal dimension of network coordination.

Importantly, the dominant neural states identified here closely align with networks previously implicated in cognitive control, attentional orienting, and stimulus-driven vigilance, supporting the biological plausibility of the data-driven decomposition. The observed abnormalities thus reflect altered temporal deployment of established networks rather than idiosyncratic or task-specific patterns.

### 5.6 Limitations and future directions

Several limitations should be acknowledged. First, although the sample size was substantial for an ERP study, the multidimensional nature of dynamic features may have limited statistical power for detecting smaller effects. Additionally, due to technical issues, behavioral data were unavailable for a small subset of participants. Although electrophysiological markers and sensitivity analyses confirmed their task compliance and the robustness of our results, the lack of overt behavioral metrics for these individuals remains a minor limitation. Second, the oddball paradigm probes a restricted set of cognitive processes; whether similar dynamic abnormalities generalize to other attentional or executive tasks remains to be determined. Third, source localization was used only to provide coarse anatomical context and should not be interpreted as precise spatial mapping. The relatively low-density EEG montage (21 channels) substantially limits the spatial resolution and reliability of inverse solutions such as sLORETA, and the estimated cortical sources should therefore be considered heuristic approximations. Future studies using high-density EEG will be necessary to more accurately characterize the cortical origins of the observed neural dynamics. Furthermore, deriving the PCA state-space from the concatenated data of all participants introduces a risk of circularity in our SVM classification. Future predictive modeling should establish the neural state templates on a strictly independent training set to prevent data leakage.

Future studies combining task-based EEG with higher spatial resolution modalities, such as fMRI or MEG, and extending dynamic analyses to longitudinal or intervention designs, will be essential for establishing the generality and clinical relevance of these findings.

## 6 Conclusions

In summary, this study demonstrates that ADHD is associated with systematic alterations in the temporal organization of neural responses during attentional processing. By revealing subtype-specific patterns of neural trajectory geometry, state stability, and transition structure, our findings provide a dynamic systems framework for understanding neurophysiological heterogeneity in ADHD. These results underscore temporal coordination of large-scale brain networks as a critical dimension of attentional dysfunction and highlight dynamic neural metrics as promising targets for future mechanistic and translational research.

## CRediT authorship contribution statement

**Yanan Zhang:** Writing – original draft, Data curation. **Tongxia Li:** Writing – original draft, Visualization, Conceptualization. **Jun Jiang:** Writing – review & editing. **Hao Li:** Writing – review & editing. **Ruidi Sun:** Writing – review & editing, Data curation. **Yunjie Li:** Writing – review & editing, Visualization, Conceptualization. **Wenqi Chen:** Writing – review & editing, Writing – original draft, Funding acquisition, Conceptualization.

## Funding

This work was supported by the Brain Science and Brain-like Intelligence Technology-National Science and Technology Major Project (No. 2025ZD0217900) and the 10.13039/501100001809National Natural Science Foundation of China (No. 32500905).

## Declaration of competing interest

The authors declare that they have no known competing financial interests or personal relationships that could have appeared to influence the work reported in this paper.

## Data Availability

The source code and sample data is publicly available online (https://github.com/ChenWQpublish/ADHD_EEG). The data supporting the findings of this study are available from the corresponding author upon reasonable request. Due to ethical and privacy considerations related to participant data, the datasets are not publicly available.

## Appendix A Supplementary data

Supplementary Data 1 Supplementary figures presenting sensitivity analyses, demographic effects, and additional neural state analyses supporting the main findings.
