# Supplementary material for: Altered temporal organization of neural response dynamics during attention processing differentiates ADHD subtypes in children
Source: Neuroimage Clin. 2026 May 23;50:104011. doi: 10.1016/j.nicl.2026.104011 (PMC13226792; doi:10.1016/j.nicl.2026.104011)
Supplement: Supplementary Data 1 — Supplementary figures presenting sensitivity analyses, demographic effects, and additional neural state analyses supporting the main findings. [file mmc1.docx]

**
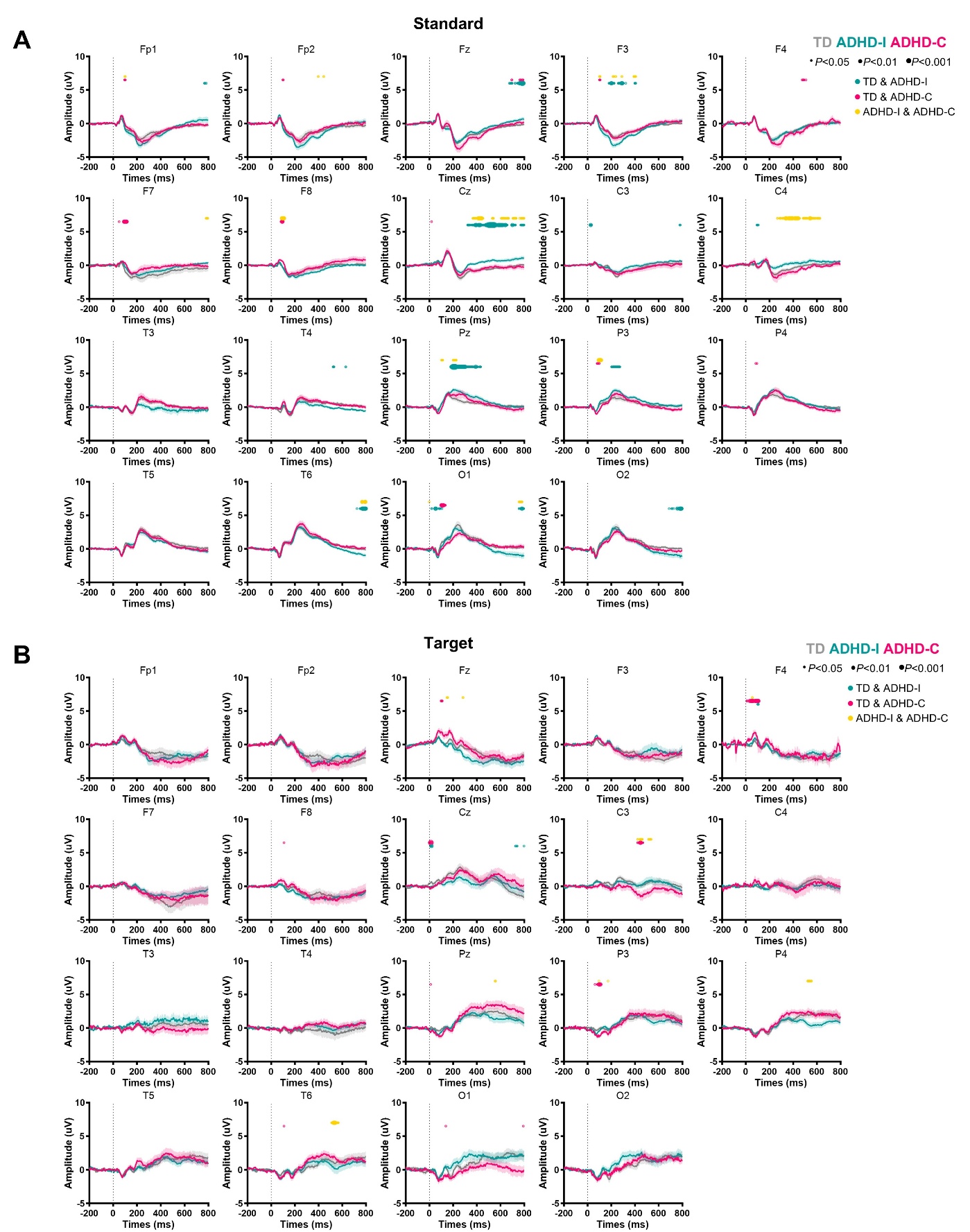
Supplementary Figure 1. Standard- and target-stimulus responses in the oddball paradigm (related to Figure 1)**

Grand-average response waveforms across all electrodes during standard and target stimuli in TD children and ADHD subgroups. Colored dots above the waveforms indicate significant group differences at the corresponding time points, with dot size reflecting significance level (Kruskal–Wallis test with Dunn–Šidák post hoc correction). Green: ADHD-I vs. TD; magenta: ADHD-C vs. TD; yellow: ADHD-I vs. ADHD-C. Data are presented as mean ± standard error.


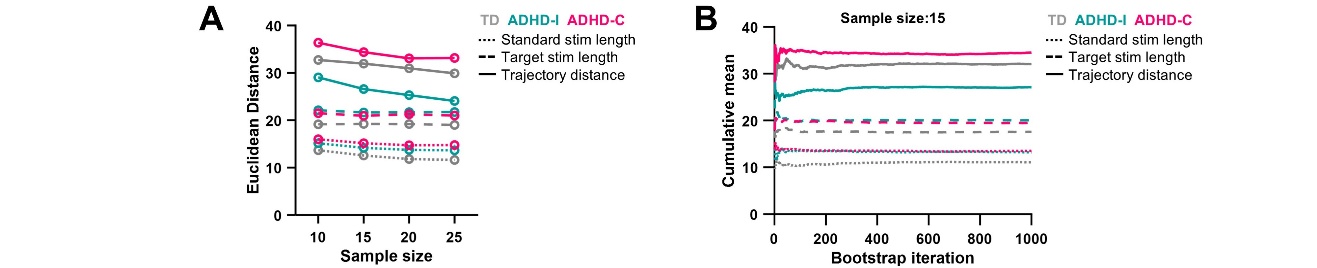
**Supplementary Figure 2. Robustness and sensitivity analysis of the bootstrap procedure for neural trajectory metrics.**

**(A)** Comparisons of group differences in neural trajectory metrics (e.g., trajectory length and between distance) across varying bootstrap subsample sizes. The statistical significance of group comparisons remained consistent across different subsample sizes (Kruskal-Wallis tests with Dunn-Šidák post hoc correction).

**(B)** Convergence of the trajectory metrics over 1000 bootstrap iterations (using N = 15). The curves illustrate that the estimations of trajectory distance and angular divergence reach a stable plateau after approximately 200 iterations.


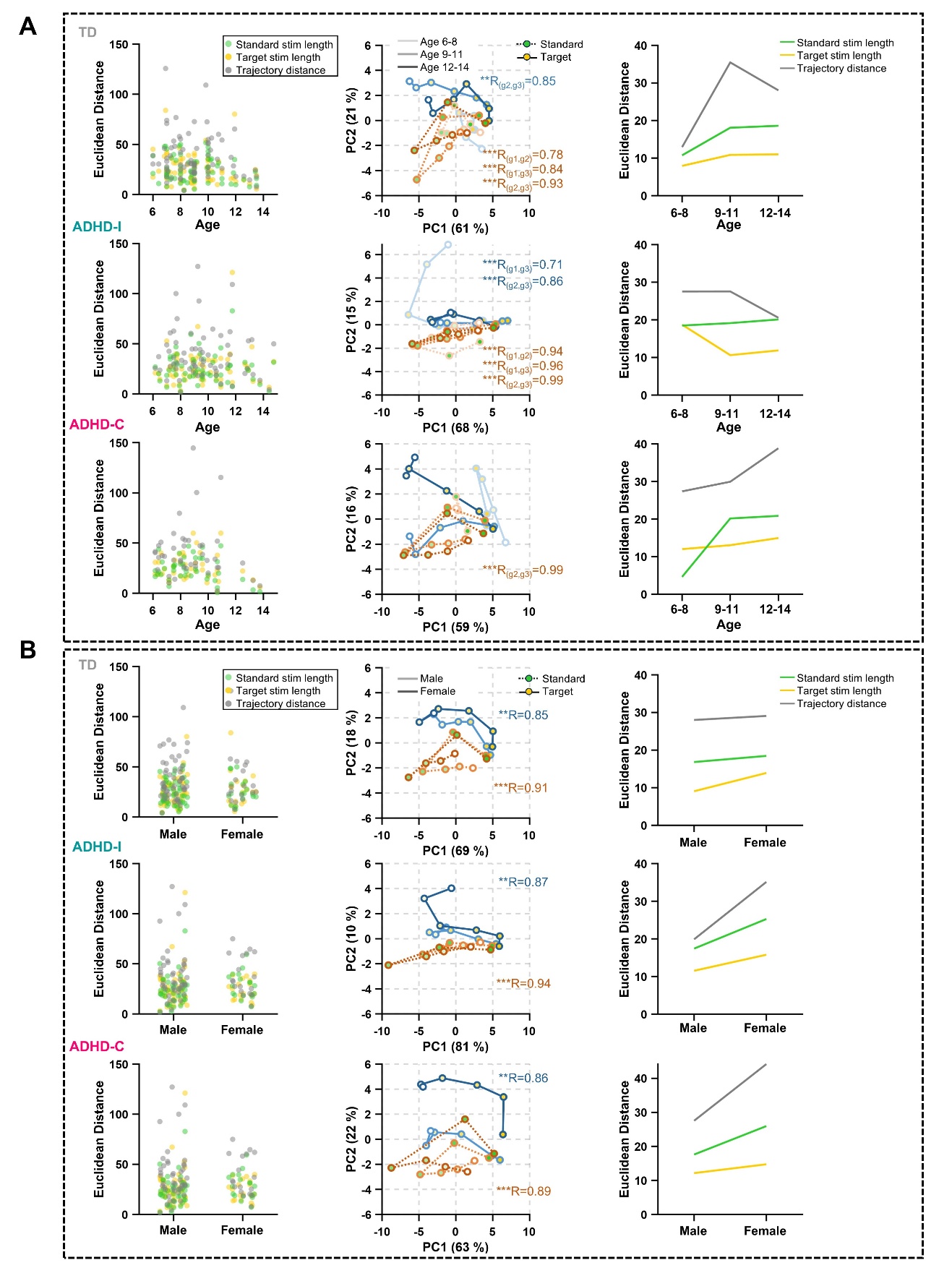
**Supplementary Figure 3. Effects of age and gender on neural trajectory dynamics in the PCA state space.**

**(A)** Age-related analysis. Rows correspond to the TD, ADHD-I, and ADHD-C groups (top to bottom). Left: Scatter plots showing the relationship between age and trajectory metrics after projecting each participant’s averaged EEG responses into the common group PCA space. Each point represents one participant; green, yellow, and gray dots indicate trajectory length for the Standard condition, trajectory length for the Target condition, and the state-space distance between the two trajectories, respectively. Middle: Neural trajectories derived from PCA within three age bins (6–8, 9–11, and 12–14 years). Orange dashed lines and blue solid lines represent Standard and Target conditions, respectively. Line color gradually darkens with increasing age. Dot color intensity decreases along the trajectory to indicate temporal progression. Right: Quantification of trajectory length and the distance between Standard and Target trajectories across the three age groups.

**(B)** Gender-related analysis. Layout and visualization are analogous to (A), but trajectories are grouped by gender. Rows correspond to TD, ADHD-I, and ADHD-C groups. The left panel shows scatter plots of trajectory metrics after projection into the common PCA space, the middle panel displays the PCA trajectories for male and female subgroups, and the right panel summarizes trajectory length and the distance between Standard and Target trajectories for the two gender groups.

**
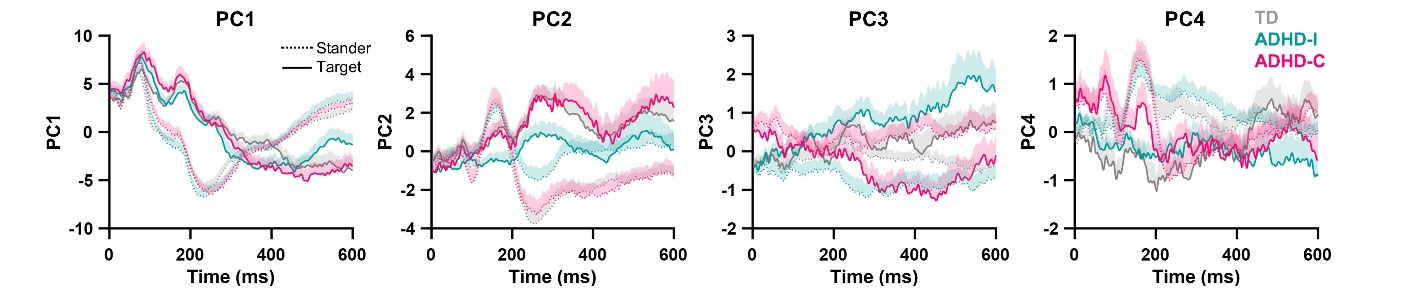
Supplementary Figure 4. Subject-level projections onto characteristic topographic states (related to Figure 4C)**

Subject-level projections onto the four characteristic topographic states (PC1–PC4) for standard and target stimuli in TD children and ADHD subgroups. Data are presented as mean ± standard error.

**
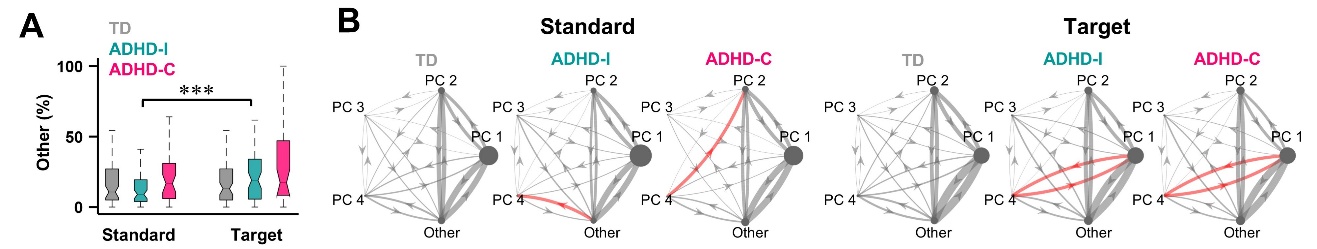
Supplementary Figure 5. State transition dynamics including the Other state across groups and conditions.**

**(A)** Proportion of time points classified as the Other state in the TD, ADHD-I, and ADHD-C groups under the Standard and Target conditions. Wilcoxon signed-rank test, ****P* < 0.001.

**(B)** Directed state transition graphs for each group. Nodes represent neural states (PC1–PC4 and Other). Edge width represents the transition probability between states, and node size reflects the probability of self-transition (self-loop). Edges showing significant differences relative to the TD group are highlighted in red (*P* < 0.05). Group differences were assessed using Kruskal-Wallis tests with Dunn’t post hoc correction.
